# Effects of fatigue on lumbar biomechanics and muscle activation across different lifting techniques: an OpenSim-based analysis

**DOI:** 10.3389/fbioe.2026.1898319

**Published:** 2026-09-29

**Authors:** Jizhai Chang, Zhicheng Li

**Affiliations:** 1 School of Humanities and Sciences, Jiangsu University of Science and Technology, Zhangjiagang, Jiangsu, China; 2 Sports Information Center, General Administration of Sport of China, Beijing, China

**Keywords:** fatigue, inverse dynamics, lifting technique, lumbar kinematics, net generalized joint moments, surface electromyography

## Abstract

**Background:**

Deadlift movements are widely used in athletic training, but how lifting technique and fatigue affect lumbar kinematics, net generalized segmental joint moments, and surface-EMG responses in Wushu athletes remains unclear.

**Purpose:**

This study compared forward-bending (FB), half-squat (HS), and deep-squat (DS) lifting to characterize lumbar kinematics, net generalized segmental joint moments, and surface-EMG responses before and after a fatiguing protocol.

**Methods:**

Fifteen male Wushu athletes performed standardized lifting tasks. Kinematic and kinetic data were analyzed using OpenSim-based scaling, inverse kinematics, and inverse dynamics with a subject-specific scaled Full-Body Lumbar Spine model to calculate lumbar kinematics and net generalized segmental joint moments. EMG processing yielded MVC-normalized muscle-activation profiles, unnormalized integrated EMG Integrated electromyographic, and median-frequency measures. Statistical analyses included repeated-measures ANOVA and paired-samples t-tests.

**Results:**

Technique- and fatigue-related differences were observed in selected lumbar kinematic, net generalized segmental joint-moment, and surface-EMG outcomes. FB showed greater lumbar flexion and higher semitendinosus IEMG, whereas HS and DS exhibited smaller lumbar excursions. HS and DS did not consistently produce lower net generalized segmental joint moments across lumbar levels. Fatigue was associated with delayed peak-moment timing and lower IEMG in several recorded muscles.

**Conclusion:**

The HS technique showed an intermediate pattern of lumbar excursion and segmental net intervertebral joint moments. These findings characterize technique-dependent patterns in lumbar kinematics and net generalized segmental joint moments.

## 1 Introduction

Lifting-related movements are fundamental components of athletic training and are widely used to enhance strength, power output, and movement efficiency. In Wushu athletes, explosive techniques such as jumping, landing, and rapid directional changes place substantial demands on trunk control and postural stability. The lumbar spine, as the central structure for force transmission between the upper and lower extremities, plays a critical role in coordinating these movements and maintaining mechanical integrity during high-intensity tasks. Previous research has demonstrated that core stability is essential for efficient movement execution and injury prevention in dynamic sports, as it provides a stable foundation for force generation and transfer (Abboud et al., 2016). In addition, the ability of the lumbar spine to maintain stability under external loading conditions is strongly influenced by neuromuscular control and trunk muscle activation patterns (McGill, 2007). However, high-load resistance exercises such as deadlifts and squats can impose substantial compressive and shear forces on the lumbar spine, increasing the mechanical demand on spinal structures (Xiao, 2025). These findings highlight the importance of understanding how different lifting strategies influence lumbar biomechanics and stability in athletic populations.

During lifting movements, the lumbar spine is subjected to substantial compressive and shear forces due to its central role in force transmission. Previous studies have shown that stoop lifting can increase lumbar shear forces by approximately 180% compared with squat-based techniques (Potvin et al., 1991). Under repetitive loading conditions, sustained spinal flexion combined with compressive forces has been associated with intervertebral disc injury, particularly when loads exceed 3,000 N (Callaghan and McGill, 2001). Importantly, lumbar loading is not uniformly distributed along the spine. Evidence indicates that the L5–S1 segment typically experiences the greatest mechanical demand (Merryweather et al., 2009), and loads exceeding 3,400 N at this level have been linked to a markedly increased risk of injury (Hoy et al., 2014). These findings highlight that lumbar mechanical stress is both magnitude-dependent and segment-specific, suggesting that evaluations based solely on global indicators may overlook critical variations in load distribution across spinal segments.

Peak lumbar compressive forces during dynamic lifting can reach 5–10 times body weight and are strongly influenced by movement posture, load distance, and lifting velocity (Kim and Zhang, 2017). Importantly, such variability is particularly relevant in athletic populations, where sport-specific training induces distinct neuromuscular control strategies and trunk stabilization patterns. For example, studies on elite martial arts athletes have demonstrated superior core stability, dynamic balance, and coordinated muscle activation compared with other athletic populations (Zhu et al., 2025). Similarly, core strength has been shown to enhance spinal stability and improve movement efficiency in Wushu athletes (Long et al., 2022). These findings suggest that lumbar loading in athletic tasks is not only influenced by external mechanical factors but also modulated by neuromuscular control. Rather than being uniformly distributed, such loads are likely redistributed across spinal segments depending on movement strategies. Therefore, understanding how different lifting techniques influence both the magnitude and distribution of lumbar loading is essential for interpreting spinal mechanical responses in athletic populations.

Further research has demonstrated that different lifting techniques lead to distinct lumbar loading patterns. Stoop lifting is generally associated with higher shear forces, whereas squat-based strategies tend to reduce shear loading but may increase compressive demand during certain phases (Khoddam-Khorasani et al., 2020). Current research primarily categorizes lifting techniques into three patterns: stoop, semi-squat, and squat (St-Vincent et al., 1989). Previous studies have further shown that these strategies result in different mechanical trade-offs, with semi-squat lifting often representing a compromise between spinal loading and movement efficiency (Kingma et al., 2001). In addition, a comprehensive review has highlighted that no single lifting technique universally minimizes spinal load, as different strategies alter the balance between compressive and shear forces (van Dieën et al., 1999). More recent evidence suggests that semi-squat lifting promotes a more neutral spinal posture and facilitates coordinated hip and knee flexion, thereby redistributing mechanical load and reducing excessive activation of the erector spinae. In contrast, stoop lifting relies more heavily on trunk musculature, leading to greater joint moments and potentially higher localized mechanical stress. Importantly, spinal stability during lifting is not determined solely by load magnitude, but also depends on neuromuscular coordination and muscle co-contraction mechanisms (Granata and Marras, 2000). These findings indicate that lifting strategies may influence not only the magnitude but also the distribution of lumbar loading, as well as the neuromuscular mechanisms underlying spinal stability.

Recent advances in muscle synergy and motor control research indicate that different lifting strategies affect not only joint mechanical loading but also the timing and contribution of muscle synergy modules. These changes may, in turn, influence movement stability and energy distribution. Therefore, evaluating lifting techniques based solely on isolated kinetic variables may be insufficient to fully capture their potential risks. Despite these advances, several limitations remain in the existing literature. Many studies have focused on static or single lifting tasks, with limited investigation of repetitive lifting under fatigued conditions. Fatigue-related biomechanical responses are task-dependent and may vary with the lifting protocol and outcome measure. Fatigue has been shown to alter movement strategies and neuromuscular control, potentially influencing lumbar loading distribution and spinal stability; however, these effects remain insufficiently explored. In addition, some studies rely on two-dimensional or simplified models, which may not adequately represent the three-dimensional dynamics of real movements. Existing research has largely emphasized external load indicators, such as ground reaction forces, with relatively less attention given to internal lumbar joint moments and neuromuscular coordination mechanisms. Although musculoskeletal modeling techniques have advanced substantially in recent years, discrepancies remain among different models in estimating spinal loads. Evidence from comparative studies indicates that compressive force predictions are generally more consistent across modeling approaches, whereas shear force estimations show greater variability and lower agreement, reflecting differences in model structure, muscle recruitment strategies, and joint representations (Ghezelbash et al., 2020).

Due to ethical and technical constraints, direct measurement of internal lumbar loads *in vivo* remains highly challenging (Sato et al., 1999). Therefore, accurately estimating internal spinal mechanical responses under conditions where direct measurement is not feasible has become a critical issue. Finite element models and traditional static biomechanical models can each provide estimates of spinal loading; however, finite element models require substantial modeling and computational resources, whereas traditional static models cannot adequately capture the inertial and time-varying characteristics of dynamic lifting tasks (Bazrgari and Shirazi-Adl, 2007). In contrast, musculoskeletal modeling approaches allow for integrated estimation of joint moments and muscle forces in complex dynamic tasks by combining kinematic, kinetic, and electromyographic data (Lloyd and Besier, 2003; Banks et al., 2023). In particular, the OpenSim platform, with its open-source architecture and extensibility, supports subject-specific, data-driven modeling and provides a physiologically meaningful framework for investigating lumbar load regulation during lifting (Seth et al., 2018). In addition, systematic reviews have highlighted that optimization-based biomechanical models and digital human modeling approaches have become important tools for analyzing lifting strategies and predicting low back injury risk (Zaman et al., 2022).

In light of the limitations identified in previous studies, the present study recruited trained male Wushu athletes to compare the kinematic, kinetic, and electromyographic characteristics of stoop, semi-squat, and squat lifting techniques under both non-fatigued and fatigued conditions. OpenSim-based scaling, inverse kinematics, and inverse dynamics were performed using the FBLS model’s joint constraints and scaled segment mass and inertial properties to calculate lumbar kinematics and net generalized segmental joint moments. The aim of this study was to characterize technique- and fatigue-related patterns in lumbar kinematics, net generalized segmental joint moments, and surface-EMG outcomes in trained male Wushu athletes. We hypothesized that these measured outcomes would vary across lifting techniques and fatigue states.

## 2 Methods

### 2.1 Participant

Fifteen trained male Wushu athletes at the Tier 3 level, corresponding to highly trained or sub-elite status, were recruited for this study. Their mean age, height, body mass, and training experience were 21.7 ± 2.3 years, 1.76 ± 0.46 m, 69.8 ± 7.4 kg, and 8.6 ± 2.1 years, respectively. A sensitivity analysis indicated that the available sample provided 80% power to detect large within-subject effects in the paired pre-to-post comparisons (dz = 0.78) and in the repeated-measures comparison across the three lifting techniques (f = 0.38); this sample size is also comparable to that used in a controlled laboratory box-lifting biomechanics study involving 15 participants (Davis and Marras, 2000). All participants were right-leg dominant. All participants had prior experience with regular deadlift training as part of their routine strength-conditioning programs. Prior to the experiment, all participants completed a standardized health screening questionnaire, including information on previous injuries, training background, recent physical condition, and cardiovascular health status.

The inclusion criteria were as follows: no history of lower-limb or low-back musculoskeletal injuries, no neurological disorders, no cardiovascular disease or other conditions affecting physical activity, and no history of surgery or severe sports-related injury within the previous 6 months. Participants who met any of these exclusion criteria were not included in the study.

All participants were instructed to refrain from vigorous physical activity for 48 h prior to testing and to maintain normal diet and sleep patterns on the day of the experiment, while avoiding alcohol and excessive caffeine intake. Before the experiment, all participants were fully informed of the study purpose, procedures, and potential risks, and provided written informed consent. The study was approved by the Ethics Committee of Capital University of Physical Education and Sports (approval number: 2024A059) and was conducted in accordance with the Declaration of Helsinki.

### 2.2 Experimental design and control conditions

A repeated-measures design was adopted to compare the biomechanical differences among three lifting techniques—forward bending (FB), half-squat (HS), and deep squat (DS)—under both non-fatigued and fatigued conditions. The three techniques were operationally defined and standardized based on the degree of lower-limb joint flexion and trunk inclination. The FB technique was characterized by lifting primarily through trunk flexion, with relatively small knee flexion and a larger trunk flexion angle. In contrast, the DS technique involved substantial hip and knee flexion while maintaining a relatively upright trunk posture. The HS technique represented an intermediate pattern, combining both trunk inclination and lower-limb flexion as a mixed movement strategy. This classification has also been applied in previous studies examining lumbar loading and joint mechanical characteristics across lifting strategies (Schmid, 2022) (Figure 1). During all trials, participants were instructed to stand with feet shoulder-width apart and parallel, and to avoid excessive trunk rotation or asymmetric loading during lifting.

**FIGURE 1:**
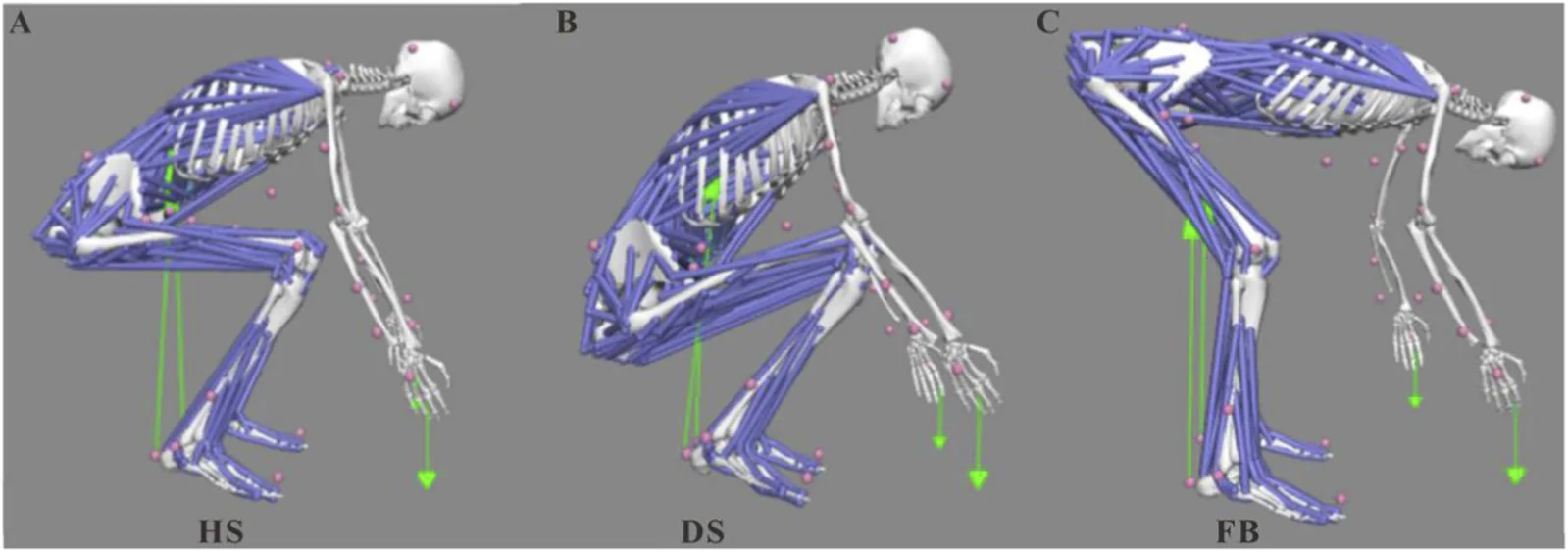
Illustration of three lifting techniques. **(A)** Half-squat lifting (HS); **(B)** Deep squat lifting (DS); **(C)** Forward bending lifting (FB).

All experiments were conducted in the same laboratory environment, with room temperature maintained at 22 °C–24 °C. All testing sessions were scheduled between 14:00 and 18:00 to minimize potential circadian effects on neuromuscular performance.

The external load was standardized as a 30-kg handled test box. The order of the three lifting techniques was randomized using a counterbalanced design. Each lifting task was performed at a controlled tempo of approximately 2 s for the lifting phase, guided by a metronome, followed by approximately 2 s of upright standing before lowering the load. Participants were instructed to maintain a consistent grip width and symmetrical hand placement across trials to ensure movement standardization. Three valid trials were collected for each technique under each condition, resulting in a total of 18 trials. A 1-min rest interval was provided between trials, and a 2-min rest interval was given between different lifting techniques to minimize non-specific fatigue.

The health-screening questionnaire covered previous musculoskeletal injury, current physical condition, cardiovascular health, and other conditions affecting physical activity; it did not include a separate maximal-strength or repeated-30-kg capacity test. Before formal data collection, participants completed a 5–10 min warm-up and familiarization trials of all three lifting techniques. During familiarization, the investigator verified that each participant could execute the prescribed task without reported pain and with acceptable technique. Testing was continuously supervised by the research team and a healthcare professional. Participants were instructed to stop immediately if they experienced pain, dizziness, unusual discomfort, or wished to discontinue; the investigator could also terminate testing if technique deteriorated or continued lifting was judged unsafe. No adverse events occurred during data collection.

### 2.3 Experimental equipment and data acquisition

All experiments were conducted in the Biomechanics Laboratory of Capital University of Physical Education and Sports. Two force plates (Kistler, Switzerland; 400 mm × 200 mm) were embedded in the center of the experimental area. The lifted object was a rigid handled test box (length × width × height: 60 cm × 40 cm × 24 cm) loaded with weight plates to a total mass of 30 kg. The weight plates were distributed evenly at the bottom of the box, and the centres of the bilateral handles were located 20 cm above the box base. The box was placed horizontally on the floor immediately in front of the force plates at the start of each trial, and participants grasped the centres of the two side handles using an opposing grip to perform the lifting task. Eight infrared cameras (OptiTrack, NaturalPoint Inc., United States) were arranged uniformly within a capture volume of approximately 3.5 m × 3.5 m × 3 m to establish a three-dimensional motion-capture environment. Kinematic, kinetic, and surface electromyography (sEMG) data were synchronously recorded throughout the experiment (Figure 2).

**FIGURE 2:**
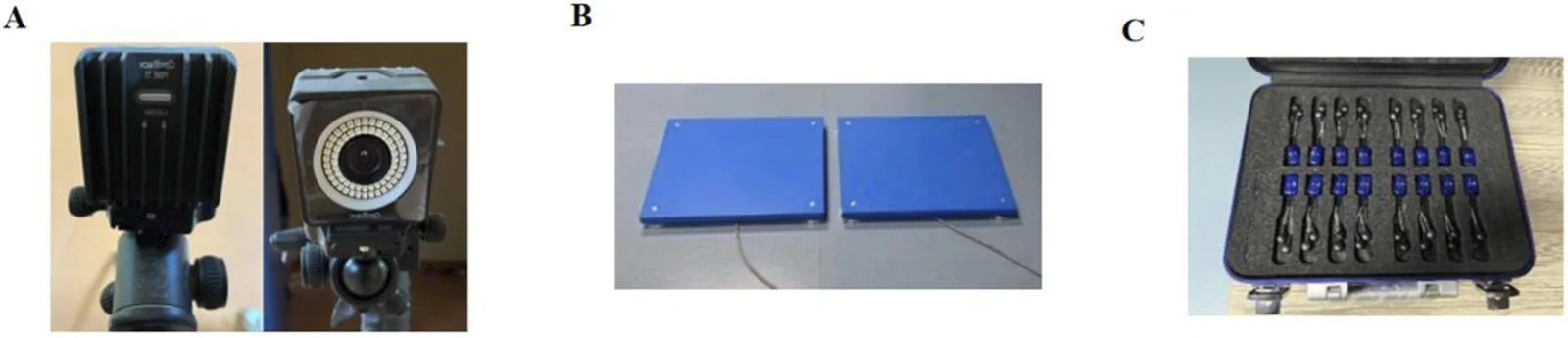
Experimental setup. **(A)** Motion capture cameras; **(B)** force plates; **(C)** EMG system.

Kinematic data were collected using a three-dimensional motion capture system (OptiTrack, NaturalPoint Inc., United States) at a sampling frequency of 200 Hz. Kinetic data were obtained using the force plates (Kistler, Switzerland) at 1,000 Hz, enabling the simultaneous recording of three-dimensional ground reaction forces (Fx, Fy, Fz) and corresponding moments. Surface electromyographic signals were recorded using a 16-channel wireless EMG system (Cometa, Italy) at 2,000 Hz. All systems were synchronized via a hardware trigger to ensure temporal alignment across data streams.

A customized 39-marker whole-body configuration was used to quantify body kinematics during the lifting task. Markers were placed bilaterally on the temple regions, posterior head regions, acromion processes, anterior and posterior superior iliac spines, lateral femoral epicondyles, lateral malleoli, heels, upper arms, elbows, forearms, wrists, and bases of the index fingers. Additional markers were placed on the C7 and T10 spinous processes, clavicle–sternum junction, lower sternum region, and right scapular region. Four technical markers were positioned on the thighs and shanks to improve segment tracking during the dynamic lifting movement. Four additional reflective markers (B1–B4) were attached to four non-coplanar corners of the test box to track its three-dimensional position and orientation. Therefore, a total of 43 markers, including 39 body markers and four box markers, were recorded during each trial. The complete marker inventory, including marker labels, anatomical locations, and functional purposes, is provided in Supplementary Table S1. Reflective markers were affixed using a commercially available general-purpose marker adhesive. Marker identity and trajectory continuity were checked in Motive during post-processing. No marker detachment occurred during the pre-fatigue trials, fatiguing protocol, or post-fatigue trials; therefore, no marker required replacement and no repeat static calibration was required. This customized marker configuration was developed for the present lifting protocol and was used for segment identification, joint-center estimation, subject-specific scaling, and inverse-kinematics analysis in OpenSim.

Sixteen sEMG channels were used to record the bilateral thoracic erector spinae, lumbar erector spinae, latissimus dorsi, gluteus maximus, semitendinosus, biceps femoris, lower rectus abdominis, and rectus femoris, with left and right homologous channels processed separately and averaged for the primary analysis because the formal lifting task was symmetrical; no inferential asymmetry analysis was performed. This selection was informed by previous electromyographic studies of squat and repetitive lifting tasks that examined trunk extensor, latissimus dorsi, abdominal, gluteal, and hamstring activity (Vakos et al., 1994; Boocock et al., 2020). Surface electrodes (Ag/AgCl disposable electrodes, approximately 10 mm in diameter, inter-electrode distance of 20 mm) were placed on the muscle belly along the direction of muscle fibers. Electrode placement followed the SENIAMguidelines to ensure consistency and reliability (Merletti et al., 2020). Prior to electrode placement, standard skin preparation procedures were performed, including shaving, alcohol cleaning, and light abrasion to reduce skin impedance and improve signal quality. Movement cadence and the fatigue protocol were controlled using a metronome to ensure consistency across trials.

### 2.4 Experimental procedure

Prior to testing, participants completed a standardized warm-up consisting of 5–10 min of light jogging and dynamic stretching, followed by familiarization trials of the three lifting techniques to ensure proper movement execution.

Before EMG acquisition, the electrode placement sites were prepared using standardized procedures, including shaving, alcohol cleaning, and light abrasion. Electrodes were then placed according to SENIAM guidelines. Maximum voluntary contraction (MVC) tests were subsequently performed to normalize the muscle-activation profiles.

MVC testing was conducted separately for each target muscle under standardized positions using isometric contractions. Participants were instructed to exert maximal effort against manual resistance or external fixation provided by the investigator. Each contraction lasted 3–5 s with standardized verbal encouragement to ensure maximal effort. Three trials were performed for each muscle, with at least 60 s of rest between trials. After excluding the first and last 0.5 s of each contraction, a 500-ms moving root-mean-square (RMS) amplitude with 50% window overlap was calculated. The largest 500-ms RMS value across the three trials was selected as the MVC reference value and used to normalize all pre- and post-fatigue trials.

During the formal trials, participants stood behind the force plates and entered the testing area upon a “start” command, positioning one foot on each force plate. The lifting task was then performed according to the prescribed technique. Each condition was repeated three times, with a 1-min rest interval between repeated trials and a 2-min rest interval between techniques; the technique order was counterbalanced across participants and retained during post-fatigue testing. Only trials with correct execution, consistent tempo, and complete data were included for further analysis.

After completing the non-fatigue condition, participants underwent the fatigue protocol. Post-fatigue testing began as soon as the fatigue protocol and immediate RPE assessment were completed and was initiated within 2 min of task termination to capture the acute post-fatigue response.

### 2.5 Fatigue protocol and criteria

To simulate fatigue during repetitive lifting tasks, a task-specific repeated lifting protocol was employed. Participants repeatedly lifted and lowered a 15-kg handled box at a fixed cadence of one complete lift every 5 s, controlled by a metronome, until the predefined task-termination criterion was met. Participants used a self-selected lifting technique; the task was not prescribed as FB, HS, or DS, and no joint-angle thresholds or technique-specific corrections were imposed. Natural self-selected lifting strategies have also been used in previous repetitive-fatigue research to examine subject-specific changes in lifting kinematics (Hawley et al., 2023). The technique adopted by each participant during the fatigue protocol was not formally classified quantitatively or qualitatively. Standardization was limited to the box mass and initial position, bilateral opposing grip, completion of the lift-and-lower cycle, and metronome cadence.

Fatigue was determined based on commonly used physiological criteria, including:

1. a rating of perceived exertion (RPE) ≥ 17 on the Borg 6–20 scale;
2. heart rate ≥85% of maximum heart rate (HRmax = 220 − age), monitored in real time using a Polar heart rate monitor;
3. inability to maintain the required lifting cadence or a noticeable decline in movement quality.

Inability to maintain the required lifting cadence or a noticeable decline in movement quality was the primary performance-based criterion for terminating the fatigue-induction task. RPE ≥17 and heart rate ≥85% of HRmax were monitored as supportive indicators of perceived exertion and internal exercise load, respectively, and were considered alongside task performance rather than as independently mandatory stopping criteria. Thus, the protocol did not require all three indicators to be met simultaneously, nor could RPE or heart rate alone trigger task termination. Post-fatigue testing began within 2 min of task termination to assess acute post-fatigue responses.

### 2.6 OpenSim analysis

OpenSim-based scaling, inverse kinematics, and inverse dynamics analyses were performed using OpenSim 4.4 (Stanford University, United States) (Seth et al., 2018). The Full-Body Lumbar Spine (FBLS) model originally developed and validated by Raabe and Chaudhari (2016) was used as the constrained multibody base model for participant-specific scaling, inverse kinematics, and inverse dynamics. The general lifting-task implementation followed that reported by Yang et al. (2025). The coupled lumbar-joint constraints were retained to represent net lumbar motion, and the model’s segment mass and inertial properties were used in the inverse-dynamics calculations.

During model scaling, the generic FBLS model was scaled to each participant using the static calibration trial and body mass. The experimental marker configuration was a customized 39-marker whole-body configuration, as described in Methods 2.3 and Supplementary Table S1. The existing virtual-marker definitions in the generic FBLS model were retained, and no model markers were manually added, repositioned, or relabeled for scaling. Segment lengths, masses, and inertial parameters were proportionally adjusted using the Scale Tool by minimizing the positional error between the experimental and model markers. The root-mean-square (RMS) error was maintained below 2 cm, with individual marker errors not exceeding 4 cm to ensure anatomical consistency.

Inverse kinematics analysis was performed using a least-squares optimization approach to estimate joint angles by minimizing the discrepancy between experimental and model marker trajectories. Kinematic data were filtered using a low-pass Butterworth filter with a cutoff frequency of 6 Hz to reduce high-frequency noise.

In this study, “lumbar flexion angle” refers to the model-derived sagittal-plane trunk/lumbar flexion–extension coordinate obtained from inverse kinematics, rather than rotation at an individual intervertebral joint. It was not calculated as the sum of the flexion–extension angles at the individual lumbar levels.

Inverse dynamics analysis was conducted by inputting ground reaction forces from the force plates together with the filtered kinematic data to calculate net generalized joint moments and related kinetic variables. No Residual Reduction Algorithm was applied.

To account for the external load during lifting, the 30-kg handled box was represented as a time-varying vertical external load. The vertical acceleration of the box was derived from the motion of the box markers, and the total vertical force was distributed equally between the left and right hand segments. The force was applied vertically downward to the hand segments and was incorporated into the inverse-dynamics calculations. Segment mass and inertial properties were scaled according to individual anthropometrics.

Raw EMG signals contained noise components such as baseline drift, power-line interference, and motion artifacts; therefore, standardized preprocessing was performed. EMG signals corresponding to the lifting phase were extracted based on synchronized kinematic data. The raw signals were band-pass filtered (10–500 Hz, fourth-order Butterworth filter) to remove low-frequency motion artifacts and high-frequency noise, and a 50 Hz notch filter was applied when necessary to eliminate power-line interference.

The filtered signals were then full-wave rectified and processed using a low-pass filter (10 Hz, fourth-order Butterworth) to obtain the linear envelope representing muscle activation over time. For the muscle-activation profiles, the linear envelope of each muscle was normalized to its MVC value to enable comparisons across participants. IEMG was calculated separately as the time integral of the unnormalized EMG voltage envelope from box lift-off to attainment of the upright, stable position during the upward lifting phase and was reported in μV·s.

Although the source FBLS model contains musculotendon actuators, no Static Optimization, Computed Muscle Control, muscle-force estimation, or joint-reaction analysis was performed in the present study. Accordingly, the OpenSim outputs were joint kinematics and net generalized joint moments only; individual muscle forces, spinal compression or shear forces, and tissue-specific loads were not estimated. Surface EMG was analyzed independently and was not used to drive or validate the OpenSim model.

### 2.7 EMG signal processing and variable calculation

Based on the processed EMG signals, the following parameters were extracted to quantify muscle activity under different lifting techniques and fatigue conditions:

1. Root-Mean-Square (RMS):

RMS was calculated to represent the amplitude of the EMG signal, reflecting the overall level of muscle activation. It was computed as:

$$RMS=\sqrt{\frac{1}{T}\int_{0}^{T}EMG{\left(t\right)}^{2}\;dt}$$

where EMG(t) is the processed EMG signal at time t, and T represents the duration of the analyzed time window.

2. Integrated EMG (iEMG):

iEMG was used to quantify the total electrical activity of the muscle over a given time period:

$$iEMG=\int_{0}^{t}\left|EMG\left(t\right)\right|dt$$

3. Median Frequency (MF):

MF was derived from the power spectral density of the EMG signal and was used as an indicator of muscle fatigue. It was defined as the frequency that divides the power spectrum into two regions with equal power:

$$\int_{0}^{MF}P\left(f\right)\;df=\frac{1}{2}\int_{0}^{f_{\max}}P\left(f\right)\;df$$

where P(f) denotes the power spectral density and f_max_ is the maximum frequency.

4. Mean Power Frequency (MPF):

MPF reflects the distribution of the EMG signal in the frequency domain and was calculated as:

$$MPF=\frac{\int_{0}^{f_{\max}}f\cdot P\left(f\right)\;df}{\int_{0}^{f_{\max}}P\left(f\right)\;df}$$

5. Muscle Activation Level:

To enable inter-subject comparison, EMG signals were normalized to the maximum voluntary contraction (MVC). The normalized muscle activation level was calculated as:

$$A=\frac{EMG_{task}}{EMGMVC}$$

where EMG task represents the EMG amplitude during the task and EMG MVC is the peak EMG value obtained during MVC testing.

### 2.8 Statistical analysis

All statistical analyses were performed using IBM SPSS Statistics version 25.0 (IBM Corp., Armonk, NY, United States). Data are presented as the mean ± standard deviation (M ± SD). The significance level was set at α = 0.05. For each participant, the three valid trials under each condition were averaged before analysis. Data normality was assessed using the Shapiro–Wilk test. Sphericity was examined using Mauchly’s test, and Greenhouse–Geisser-corrected results were reported when the sphericity assumption was violated. Each kinematic, kinetic, and EMG outcome was analyzed using a 3 × 2 two-way repeated-measures ANOVA, with lifting technique (HS, DS, and FB) and fatigue state (Pre and Post) as within-subject factors. The main effects of lifting technique and fatigue state and their interaction were examined. Partial eta squared (ηp^2^) was reported as the effect-size measure.

When a significant main effect of lifting technique or a significant interaction was identified, pairwise comparisons among the three techniques were conducted separately within the Pre- and Post-fatigue conditions using Bonferroni-adjusted P values. Significant interactions were further examined using simple-effect analyses. Planned Pre–Post comparisons within each technique were performed using paired-samples t-tests.

## 3 Result

### 3.1 Median frequency of EMG during fatigue induction

Participants completed 162 ± 34 complete lift-and-lower cycles before task termination (range: 105–220 cycles). Median frequency (MF) decreased from the beginning to the end of the fatigue-induction task. Paired-samples t-tests showed significant pre-to-post decreases in MF for the thoracic erector spinae (P < 0.01), lumbar erector spinae (P < 0.01), latissimus dorsi (P < 0.01), gluteus maximus (P < 0.001), semitendinosus (P < 0.05), biceps femoris (P < 0.01), lower rectus abdominis (P < 0.01), and rectus femoris (P < 0.01). These spectral changes in the recorded EMG signals were consistent with the fatigue induced by the repetitive lifting protocol (Figure 3).

**FIGURE 3:**
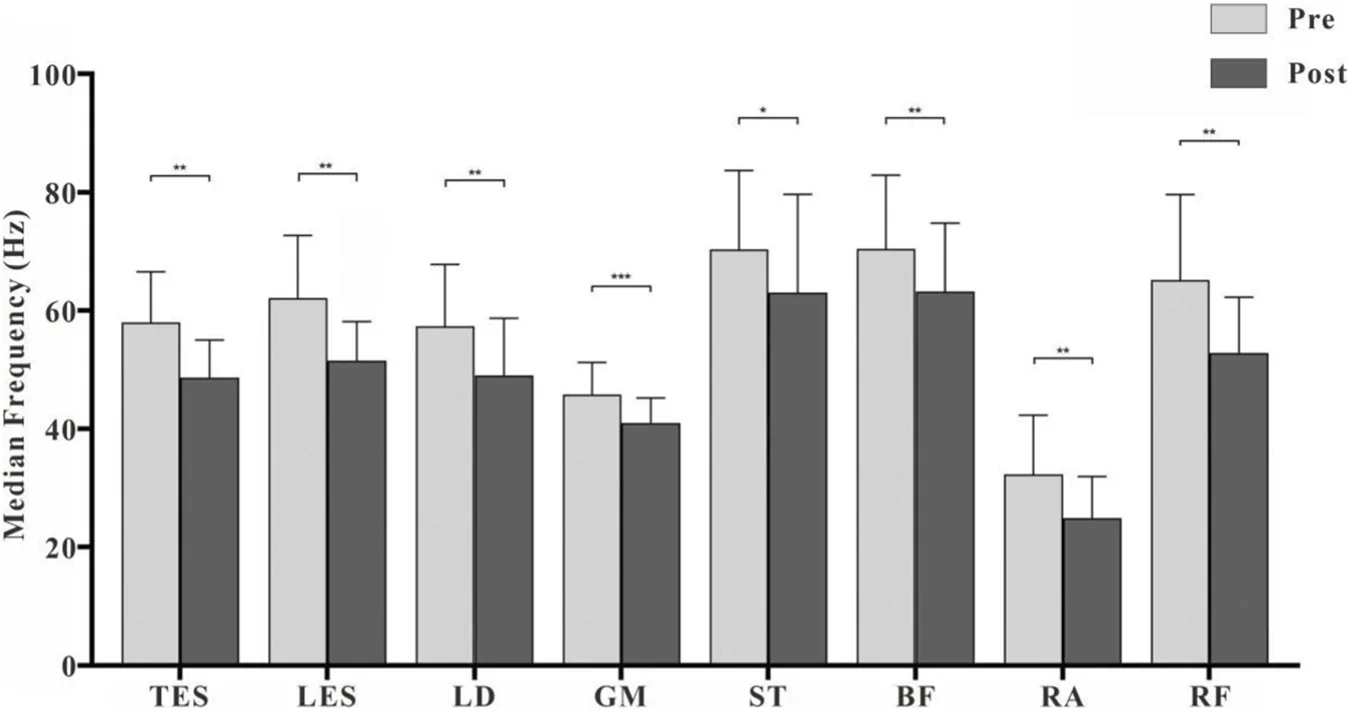
Comparison of median frequency (MF) of EMG signals before and after fatigue during the fatigue protocol. Bars and error bars represent the mean and standard deviation, respectively. *P < 0.05, **P < 0.01, and ***P < 0.001.

### 3.2 Kinematic characteristics of the lumbar joint across lifting techniques before and after fatigue

#### 3.2.1 Lifting time

A two-way repeated-measures ANOVA revealed significant main effects of lifting technique, F (2, 28) = 9.25, P < 0.001, ηp^2^ = 0.398, and fatigue state, F (1, 14) = 6.13, P = 0.027, ηp^2^ = 0.305. The Technique × Fatigue interaction was not significant, F (2, 28) = 0.01, P = 0.988, ηp^2^ = 0.001.

Before fatigue induction, the completion time for FB (1.30 ± 0.24 s) was significantly longer than that for HS (1.13 ± 0.19 s, Bonferroni-adjusted P < 0.001) and DS (1.16 ± 0.22 s, Bonferroni-adjusted P = 0.005). No significant difference was observed between HS and DS (adjusted P = 0.369) (Figure 4).

**FIGURE 4:**
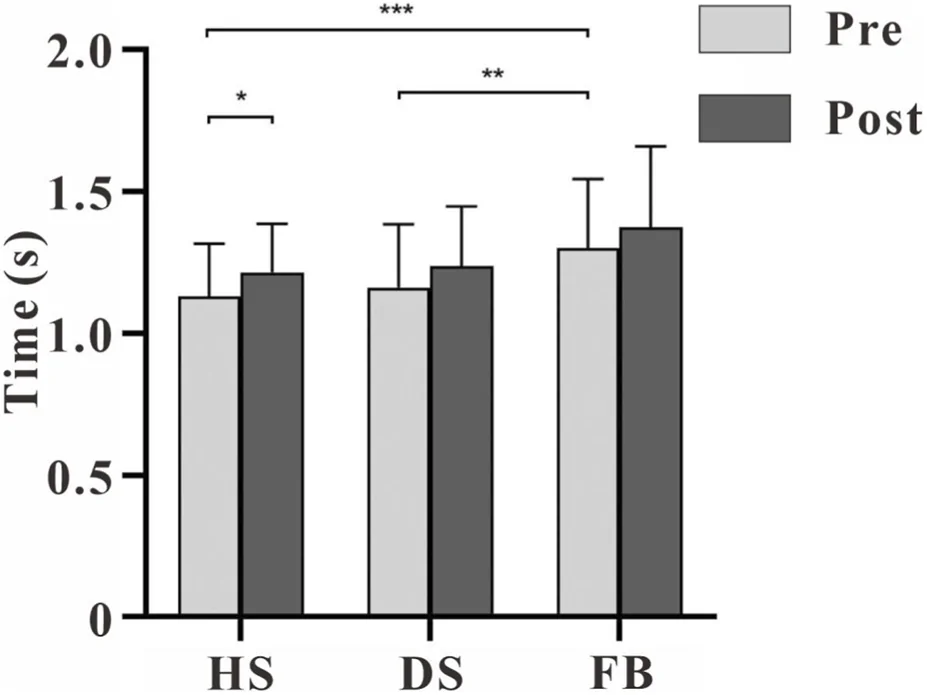
Time required for the three lifting techniques before and after fatigue (unit: s). Bars and error bars represent the mean and standard deviation, respectively. *P < 0.05, **P < 0.01, and ***P < 0.001.

Following fatigue induction, the mean completion times increased numerically to 1.21 ± 0.17 s for HS, 1.24 ± 0.21 s for DS, and 1.37 ± 0.29 s for FB. The differences among the three lifting techniques did not reach statistical significance after Bonferroni correction (all adjusted P ≥ 0.222). Paired-samples t-tests demonstrated a significant increase in completion time for HS after fatigue induction, t (14) = 2.36, P = 0.033. In contrast, the Pre- Post differences were not significant for DS, t (14) = 1.32, P = 0.208, or FB, t (14) = 1.18, P = 0.256.

#### 3.2.2 Changes in the lumbar flexion angle

Figure 5 presents the time-normalized lumbar flexion-angle trajectories before and after fatigue for the three deadlift techniques. The Pre- and Post-fatigue curves showed broadly similar overall profiles within each technique. HS and DS exhibited comparable overall lumbar flexion patterns, whereas FB showed a greater flexion excursion. According to the adopted angular sign convention, greater lumbar flexion was represented by more negative values. The absolute peak flexion magnitude was approximately 40° for HS and DS and approximately 78°–80° for FB. FB therefore exhibited a substantially greater lumbar range of motion than HS and DS, whereas the lumbar flexion angle approached 0° under all conditions (Table 1).

**FIGURE 5:**
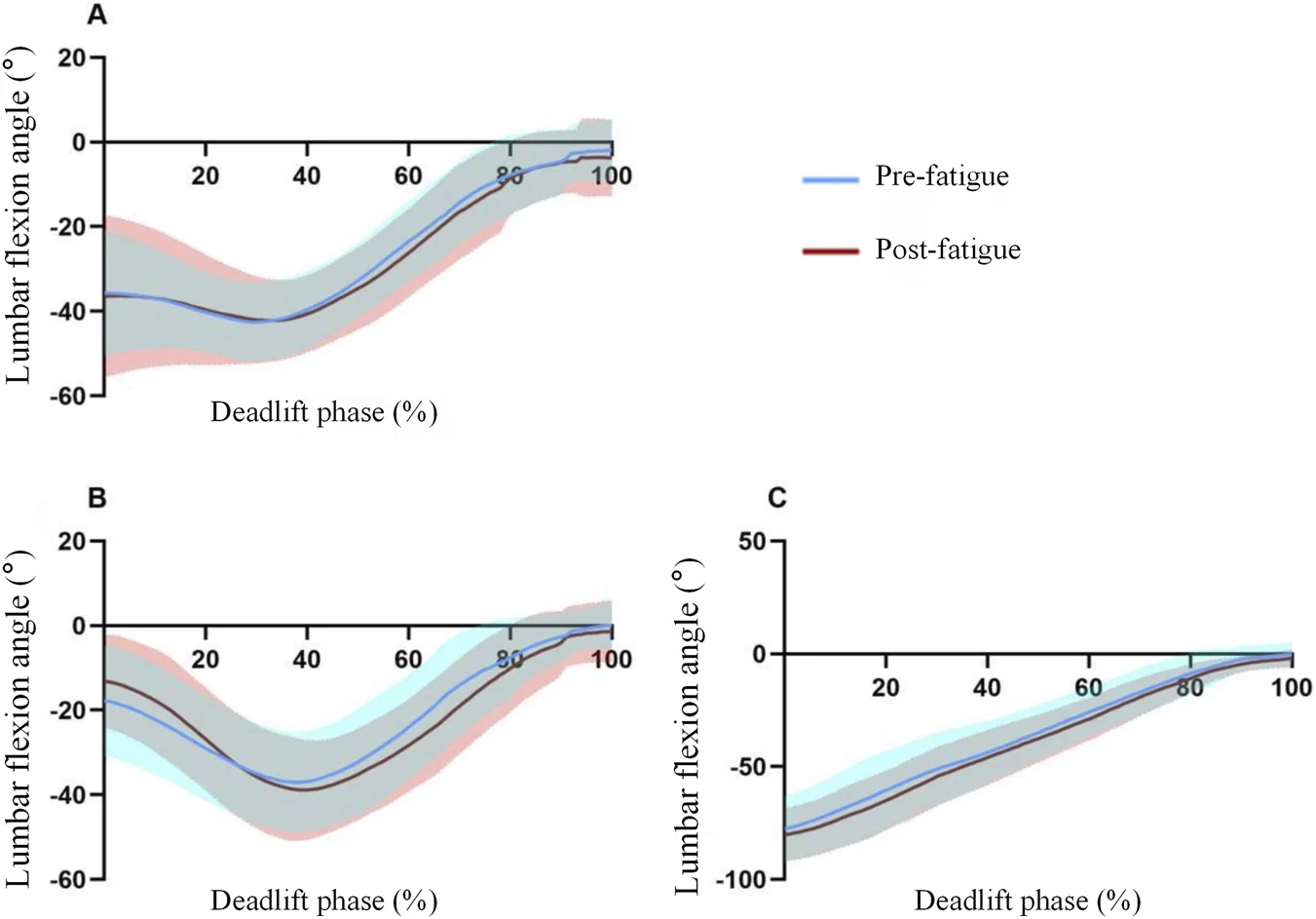
Time-normalized lumbar flexion-angle trajectories before and after fatigue for **(A)** HS (half squat), **(B)** DS (deep squat), and **(C)** FB (forward bending).

**TABLE 1:** Comparison of lumbar flexion angle across three lifting techniques before and after fatigue (unit: °).

Variable	HS pre	HS post	DS pre	DS post	FB pre	FB post
Minimum (°)	−1.82	−3.61	0.02	−1.38	−0.29	−1.90
Phase of minimum (%)	100	94	100	100	100	100
Maximum (°)	−42.56	−42.18	−37.02	−38.82	−77.56	−80.15
Phase of maximum (%)	30	32	38	40	0	0
Range of motion (°)	40.74	38.57	37.04	37.44	77.27	78.25

All values are derived from averaged fitted curves. HS, half squat; DS, deep squat; FB, forward bending.

The two-way repeated-measures ANOVA showed a significant main effect of lifting technique on lumbar ROM, F (2, 28) = 25.24, P < 0.001, ηp^2^ = 0.643. Neither the main effect of fatigue state, F (1, 14) = 0.13, P = 0.729, ηp^2^ = 0.009, nor the Technique × Fatigue interaction, F (2, 28) = 1.03, P = 0.370, ηp^2^ = 0.069, was significant.

Before fatigue induction, lumbar ROM during FB (77.27° ± 18.00°) was significantly greater than during HS (40.74° ± 13.00°) and DS (37.04° ± 13.00°), with both Bonferroni-adjusted P values < 0.001. Similarly, after fatigue induction, lumbar ROM during FB (78.25° ± 17.00°) remained significantly greater than during HS (38.57° ± 14.00°) and DS (37.44° ± 14.00°), with both adjusted P values < 0.001. No significant difference was found between HS and DS either before fatigue (adjusted P = 0.388) or after fatigue (adjusted P = 0.548). In addition, none of the three lifting techniques showed a significant Pre–Post change in lumbar ROM (all P ≥ 0.338) (Figure 6).

**FIGURE 6:**
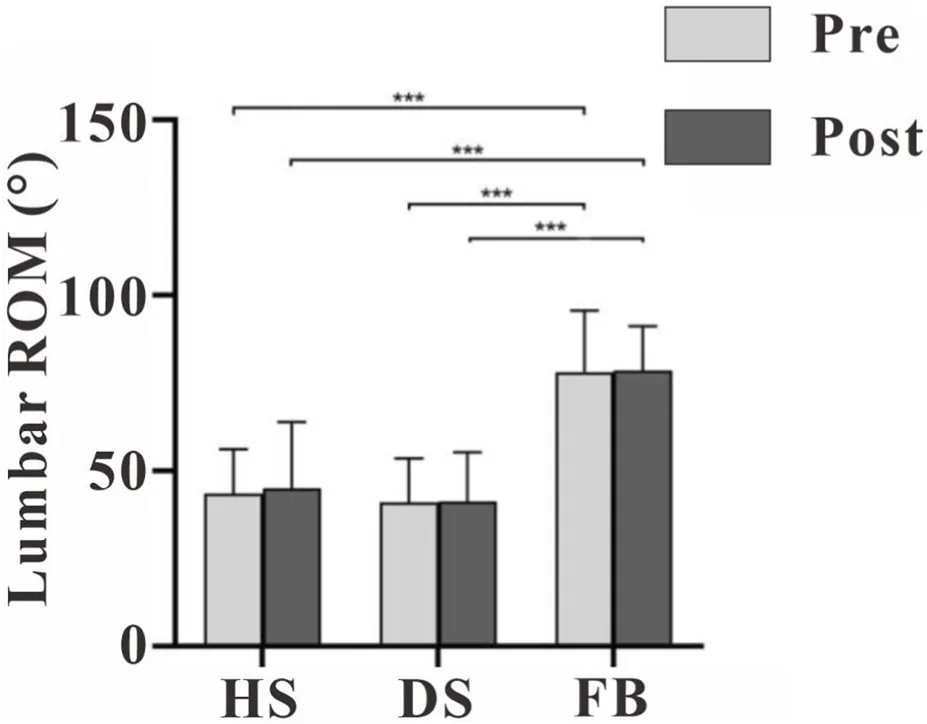
Maximum lumbar joint range of motion during three lifting techniques before and after fatigue induction (unit: °): HS (half squat), DS (deep squat), and FB (forward bending). Bars and error bars represent the mean and standard deviation, respectively. ***P < 0.001.

### 3.3 Kinetic characteristics of the lumbar joint across lifting techniques before and after fatigue induction

Peak lumbar joint moments generally decreased from the lower to the upper lumbar segments across all lifting techniques. The highest values were observed at L4–L5, followed by L3–L4, L2–L3, and L1–L2.

At L4–L5 (Figure 7A), the pre- and post-fatigue values were 3.78 ± 0.30 and 3.69 ± 0.30 N m/kg for HS, respectively. The corresponding values were 3.59 ± 0.29 and 3.71 ± 0.31 N m/kg for DS and 3.71 ± 0.36 and 3.74 ± 0.34 N m/kg for FB. A significant main effect of lifting technique was observed, F (2, 28) = 4.58, P = 0.019, ηp^2^ = 0.247. The Technique × Fatigue interaction was also significant, F (2, 28) = 4.53, P = 0.020, ηp^2^ = 0.245. Before fatigue, HS produced a significantly greater peak moment than DS (P = 0.007).

**FIGURE 7:**
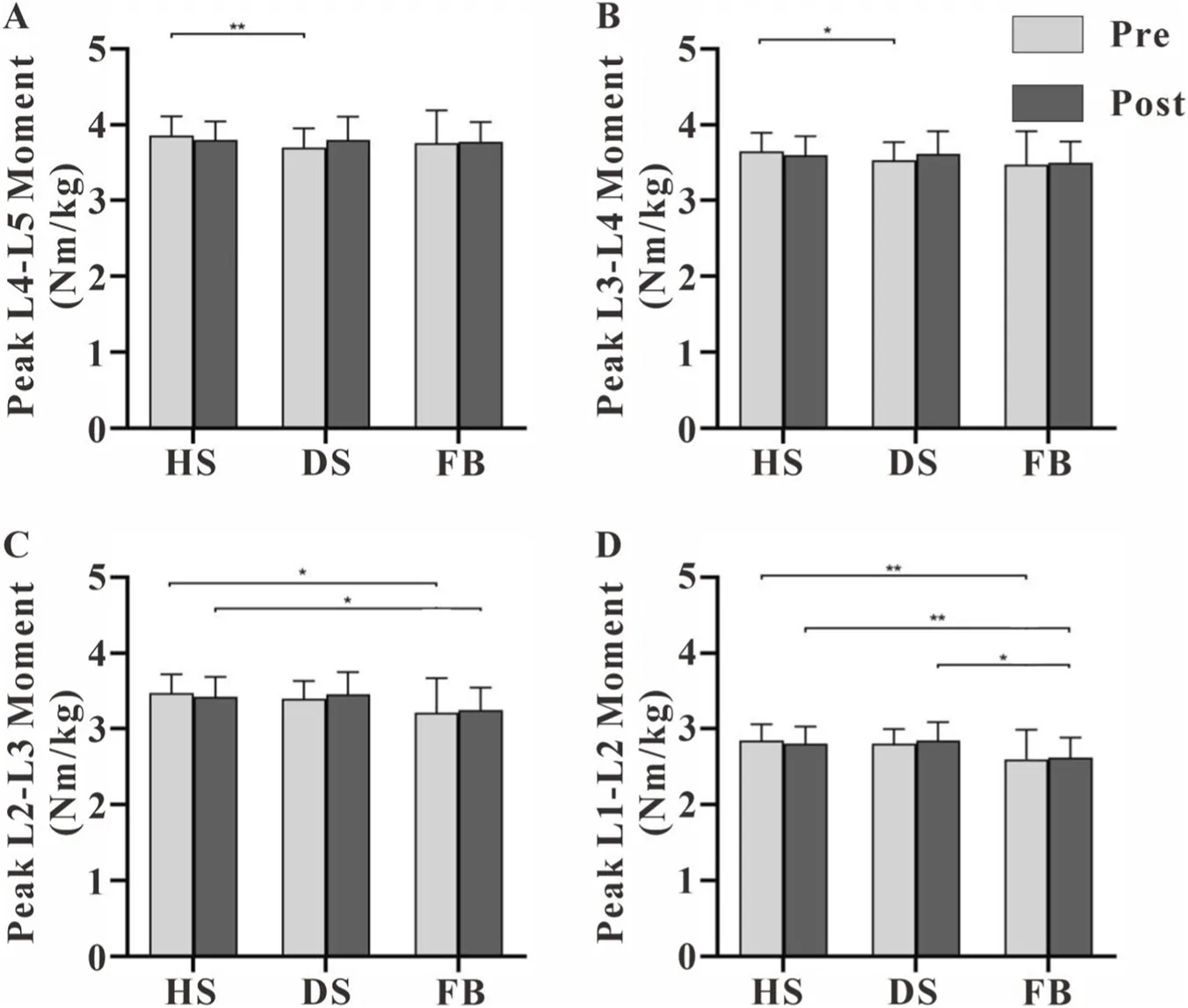
Peak joint moments of the lumbar spine (N m/kg). **(A)** Comparison of peak joint moment at L4–L5 before and after fatigue induction; **(B)** comparison at L3–L4; **(C)** comparison at L2–L3; **(D)** comparison at L1–L2. Light colors represent the pre-fatigue condition, and dark colors represent the post-fatigue condition. Bars and error bars represent the mean and standard deviation, respectively. *P < 0.05, **P < 0.01.

At L3–L4 (Figure 7B), the pre- and post-fatigue values were 3.60 ± 0.28 and 3.52 ± 0.29 N m/kg for HS, respectively. The corresponding values were 3.50 ± 0.27 and 3.60 ± 0.30 N m/kg for DS and 3.45 ± 0.31 and 3.45 ± 0.30 N m/kg for FB. The main effect of lifting technique was significant, F (2, 28) = 4.95, P = 0.014, ηp^2^ = 0.261. Before fatigue, HS produced a significantly greater peak moment than DS (P = 0.049).

At L2–L3 (Figure 7C), the pre- and post-fatigue values were 3.43 ± 0.26 and 3.36 ± 0.27 N m/kg for HS, respectively. The corresponding values were 3.36 ± 0.27 and 3.43 ± 0.28 N m/kg for DS and 3.20 ± 0.34 and 3.20 ± 0.32 N m/kg for FB. A significant main effect of lifting technique was detected, F (2, 28) = 7.40, P = 0.003, ηp^2^ = 0.346. HS produced significantly greater peak moments than FB both before (P = 0.028) and after fatigue (P = 0.035).

At L1–L2 (Figure 7D), the pre- and post-fatigue values were 2.81 ± 0.23 and 2.76 ± 0.24 N m/kg for HS, respectively. The corresponding values were 2.75 ± 0.23 and 2.82 ± 0.25 N m/kg for DS and 2.60 ± 0.35 and 2.60 ± 0.31 N m/kg for FB. The main effect of lifting technique was significant, F (2, 28) = 8.41, P = 0.001, ηp^2^ = 0.375. HS produced significantly greater peak moments than FB both before (P = 0.009) and after fatigue (P = 0.006). After fatigue, DS also produced a significantly greater peak moment than FB (P = 0.049).

No significant main effect of fatigue was detected at any lumbar level (all P > 0.05). The Technique × Fatigue interactions at L3–L4, L2–L3, and L1–L2 were also not significant (all P > 0.05). Apart from the differences reported above, neither the remaining pairwise comparisons nor the pre-to post-fatigue comparisons within each lifting technique reached statistical significance (all P > 0.05).

### 3.4 Integrated EMG across muscles and lifting techniques before and after fatigue

The integrated electromyographic values of all muscles under HS, DS, and FB conditions before and after fatigue induction are presented in Figures 8–10.

**FIGURE 8:**
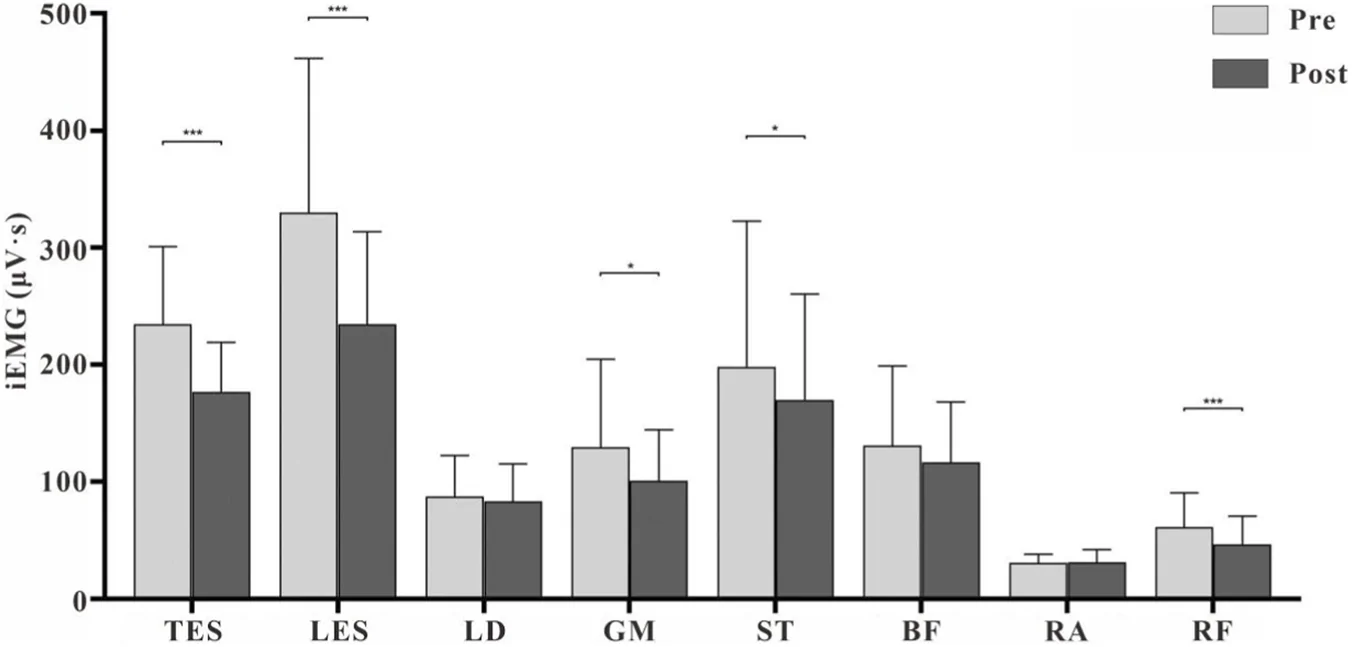
Integrated electromyographic (IEMG) values of each muscle under the HS (half squat) lifting technique before and after fatigue induction (unit: μV s). Bars and error bars represent the mean and standard deviation, respectively. *P < 0.05, **P < 0.01, and ***P < 0.001.

**FIGURE 9:**
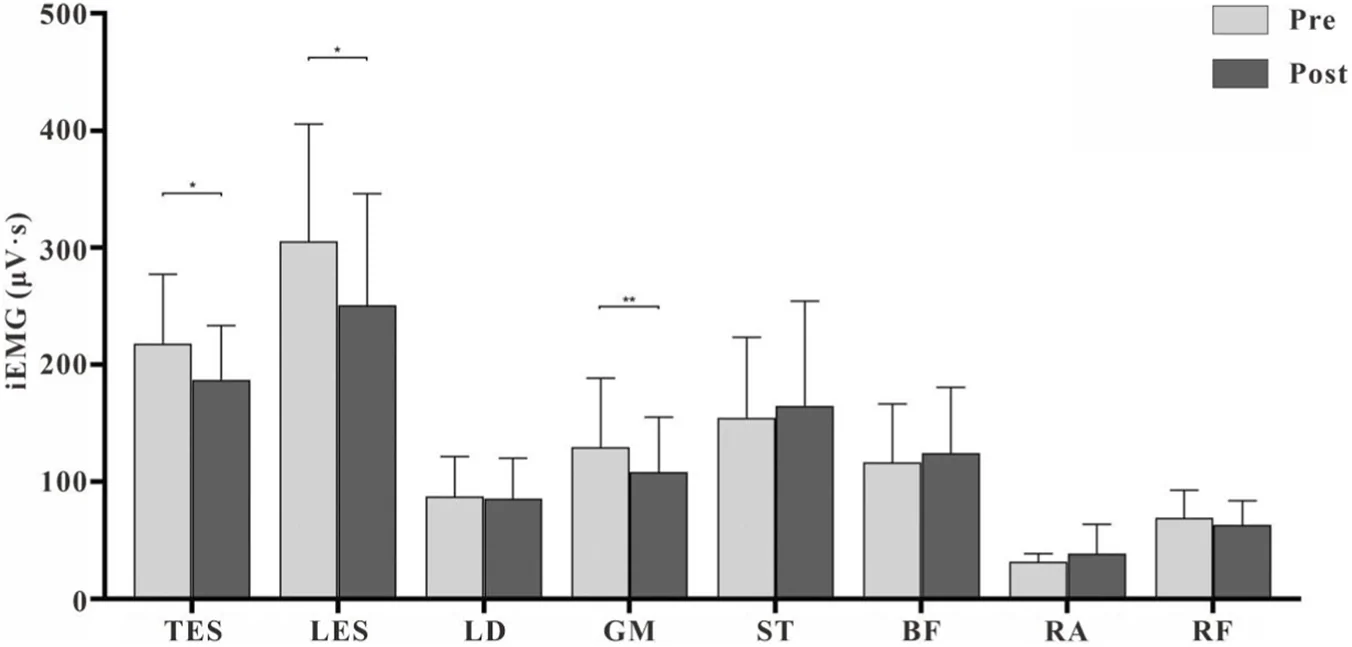
Integrated electromyographic (IEMG) values of each muscle under the DS (deep squat) lifting technique before and after fatigue induction (unit: μV·s). Bars and error bars represent the mean and standard deviation, respectively. *P < 0.05, **P < 0.01.

**FIGURE 10:**
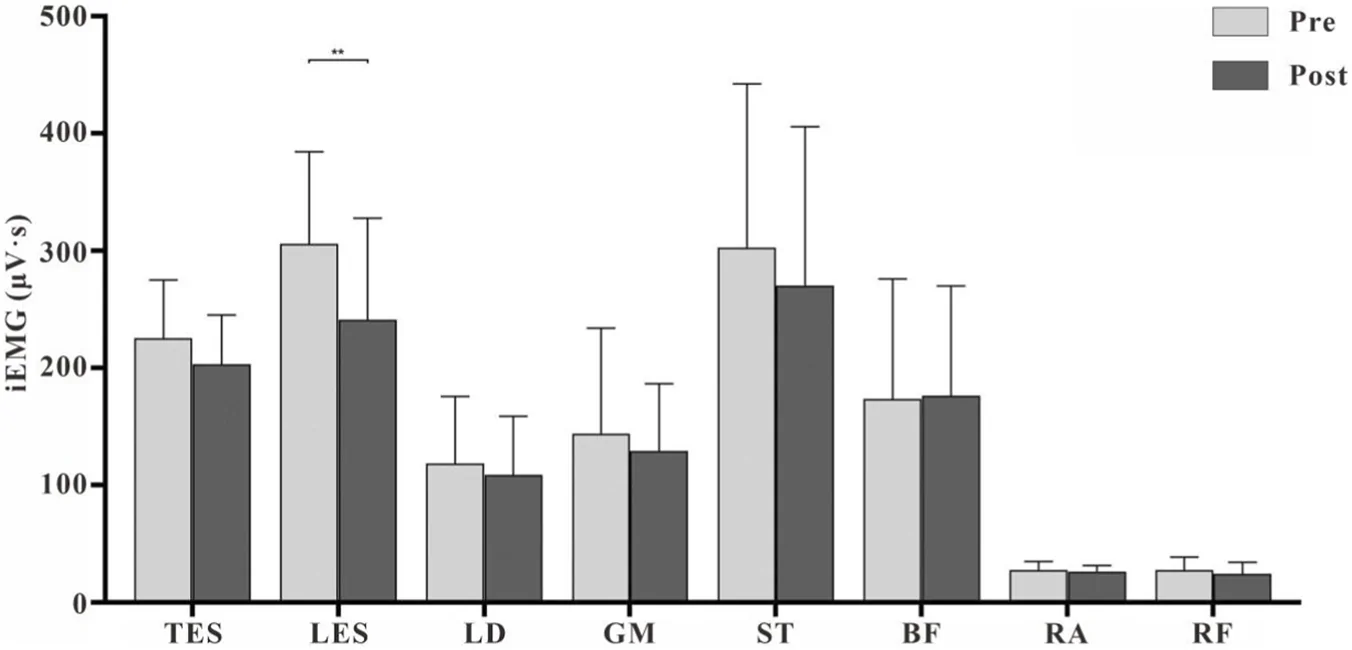
Integrated electromyographic (IEMG) values of each muscle under the FB (forward bending) lifting technique before and after fatigue induction (unit: μV·s). Bars and error bars represent the mean and standard deviation, respectively. **P < 0.01.

Across all lifting techniques, most muscles exhibited lower IEMG values following fatigue induction. Under HS, notable reductions were observed in the thoracic erector spinae (234.61 ± 66.19 vs. 177.11 ± 41.95 μV·s), lumbar erector spinae (329.76 ± 132.02 vs. 234.55 ± 78.97 μV·s), gluteus maximus (129.62 ± 75.02 vs. 101.01 ± 43.42 μV·s), semitendinosus (198.25 ± 124.16 vs. 170.14 ± 90.30 μV·s), and rectus femoris (61.22 ± 29.53 vs. 46.47 ± 24.16 μV·s).

Under DS, similar reductions were observed in the thoracic erector spinae (218.20 ± 58.94 vs. 187.12 ± 46.17 μV·s), lumbar erector spinae (305.43 ± 100.15 vs. 251.02 ± 95.06 μV·s), and gluteus maximus (129.72 ± 58.80 vs. 108.50 ± 46.84 μV·s).

Under FB, decreases were observed in the lumbar erector spinae (305.67 ± 78.75 vs. 240.65 ± 86.96 μV·s), semitendinosus (302.75 ± 139.65 vs. 270.39 ± 135.43 μV·s), and gluteus maximus (143.81 ± 90.06 vs. 129.13 ± 57.27 μV·s), while other muscles showed relatively smaller changes.

Paired-sample t-test results indicated that, under HS, significant differences between pre- and post-fatigue conditions were observed in the thoracic erector spinae (P < 0.001), lumbar erector spinae (P < 0.001), gluteus maximus (P < 0.05), semitendinosus (P < 0.05), and rectus femoris (P < 0.001). Under DS, significant differences were found in the thoracic erector spinae (P < 0.05), lumbar erector spinae (P < 0.05), and gluteus maximus (P < 0.01). Under FB, only the lumbar erector spinae showed a significant difference (P < 0.01).

Figure11 shows the comparison of IEMG values across muscles under three lifting techniques before fatigue induction.

**FIGURE 11:**
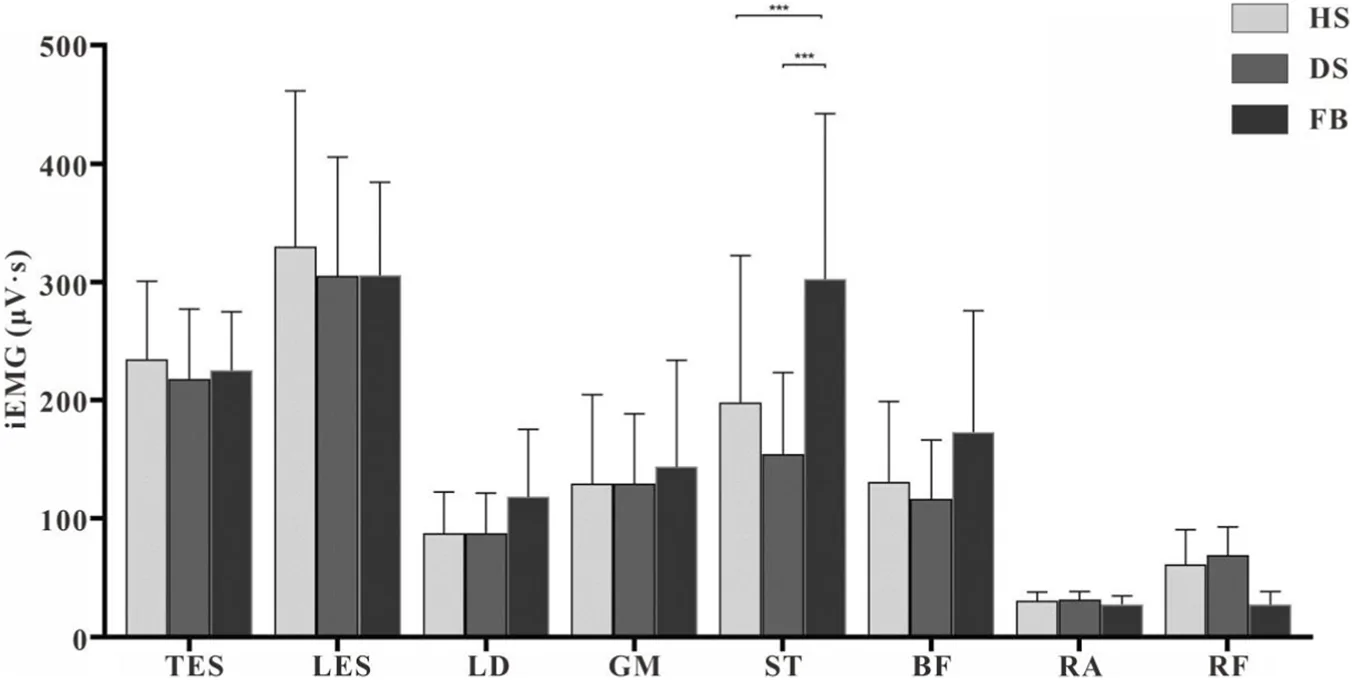
Integrated electromyographic (IEMG) values of each muscle across three lifting techniques before fatigue induction (unit: μV·s). Bars and error bars represent the mean and standard deviation, respectively. ***P < 0.001.

Two-way repeated-measures ANOVA revealed a significant main effect of lifting technique only for the semitendinosus (ST), F (2, 28) = 13.53, P < 0.001, ηp^2^ = 0.491. Bonferroni-adjusted comparisons showed that, before fatigue induction, ST IEMG during FB was significantly greater than during HS (adjusted P < 0.001) and DS (adjusted P < 0.001), whereas the difference between HS and DS was not significant. No significant differences among lifting techniques were observed for ST after fatigue induction. Although BF showed numerically higher mean IEMG during FB, its main effect of lifting technique and all pairwise comparisons were not significant. The technique effects and pairwise comparisons for TES, LES, LD, GM, RA, and RF were likewise nonsignificant (all P > 0.05). No significant Technique × Fatigue interaction was detected for any recorded muscle (all P > 0.05) (Figure 10).

## 4 Discussion

The present study systematically investigated the biomechanical and neuromuscular characteristics of three lifting techniques (HS, DS, and FB) under pre- and post-fatigue conditions using OpenSim-based inverse kinematics and inverse dynamics analyses together with independent surface-EMG analysis. The findings partially supported the initial hypotheses. Specifically, compared with FB, HS and DS did not consistently exhibit lower net generalized lumbar joint moments across all segments, although segment-specific moment patterns differed among techniques. Fatigue was associated with delayed peak-moment timing and lower IEMG in several recorded muscles. Accordingly, the findings are interpreted in terms of the measured lumbar kinematics, net generalized segmental joint moments, and EMG-derived outcomes.

### 4.1 Biomechanical differences among lifting techniques

Differences among lifting techniques in the present study were primarily reflected in lumbar kinematics, net generalized segmental joint moments, and IEMG of the recorded muscles. The FB technique was characterized by greater lumbar flexion, whereas HS and DS exhibited relatively smaller lumbar excursions. This pattern is generally consistent with previous literature indicating that stoop- and squat-based lifting strategies represent distinct biomechanical solutions rather than a simple dichotomy of “unsafe” versus “safe” movements (van Dieën et al., 1999; Washmuth et al., 2022).

Musculoskeletal modeling studies have further demonstrated that stoop lifting is often associated with increased shear-oriented loading, whereas squat lifting may redistribute load toward compressive components and increase lower-extremity demand (Bazrgari et al., 2007; von Arx et al., 2021). In the present study, the reduced lumbar flexion observed in HS and DS may reflect a greater contribution from the hip and knee joints, thereby facilitating load transfer to the lower extremities rather than concentrating mechanical demand within the lumbar spine (Tröster et al., 2022; Kingma et al., 2010), hip and knee joint moments were not analyzed in the present study. Therefore, the present findings cannot be interpreted as evidence of load transfer to the lower extremities or reduced internal lumbar loading.

The greater semitendinosus IEMG observed under FB indicates a technique-related difference in one recorded posterior-thigh muscle during the upward lifting phase. Previous studies have shown that stoop lifting may alter lumbopelvic rhythm and increase reliance on hamstring–lumbar interactions, particularly under loaded conditions (Iwasaki et al., 2014). Moreover, experimental comparisons have reported physiological and biomechanical differences among lifting techniques (Leskinen et al., 1983).

Consistent with this perspective, HS in the present study did not consistently exhibit lower lumbar joint moments than DS or FB across all segments. Instead, HS displayed an intermediate pattern in the measured lumbar flexion and net generalized segmental joint moments. Such findings align with previous work indicating that spinal loading during lifting emerges from the interaction between passive tissue properties, active muscle forces, and whole-body coordination strategies (Bazrgari and Shirazi-Adl, 2007). Taken together, the present results should be interpreted as differences in the measured lumbar kinematics, net generalized segmental joint moments, and IEMG of the recorded muscles, rather than as direct evidence of differences in internal spinal loading, lower-extremity load redistribution, or spinal protection.

### 4.2 Fatigue-related changes in joint-moment timing and muscle activity

A key finding of the present study was that fatigue did not result in a substantial increase in net generalized lumbar joint moments, but was associated with delayed peak-moment timing and lower IEMG in several recorded muscles. These findings describe changes in the timing of the measured net generalized lumbar joint moments and in IEMG of the recorded muscles; they do not establish changes in internal spinal loading or a specific neuromuscular control mechanism. Early studies on repetitive lifting demonstrated that fatigue of the erector spinae can modify spinal loading and increase bending moments under certain conditions (Dolan and Adams, 1998; Potvin and Norman, 1993). However, subsequent work has indicated that fatigue-related responses are more complex and may involve changes in coordination patterns and load-sharing mechanisms rather than uniform increases in joint loading (van Dieën et al., 1996; Sparto et al., 1997).

In this context, the delayed occurrence of peak joint moments observed in the present study may reflect a fatigue-induced reorganization of movement timing. This interpretation is consistent with evidence that fatigue can alter neuromuscular control of spinal stability and reduce the dynamic stability of the trunk (Granata et al., 2004; Granata and Gottipati, 2008). In addition, adaptations in trunk muscle recruitment and coactivation have been reported as compensatory responses aimed at maintaining spinal stability under fatigued conditions (Granata and Orishimo, 2001). However, spinal stability and muscle-force-related coactivation were not quantified in the present study; therefore, the observed timing changes should not be interpreted as direct evidence of altered spinal stability.

More recent studies have further shown that fatigue can influence cumulative lumbar loading, modify intervertebral kinematics, and alter local dynamic stability during repetitive lifting tasks (Kazemi et al., 2024; Larson and Brown, 2022; Asgari et al., 2017). Recent work also indicates that fatigue-related biomechanical adaptations are task-dependent: repetitive lifting may be accompanied by altered movement behavior (Ratke et al., 2025), whereas lifting kinematics and model-estimated low-back loads may remain relatively unchanged in other protocols (Tabasi et al., 2023). A recent systematic review of experienced lifters likewise reported more consistent effects of fatigue on movement velocity and power than on force production and joint mechanics (Bakhshinejad et al., 2025). Fatigue does not necessarily produce a uniform directional change in surface-EMG amplitude during dynamic lifting. Experimental evidence indicates that fatigue-related changes in EMG amplitude and frequency during dynamic contractions depend on muscle length and contraction velocity, while methodological reviews emphasize that task characteristics and the selected EMG metric also affect interpretation (Potvin, 1997; Vigotsky et al., 2017). Consequently, results based on peak amplitude, RMS, and phase-integrated IEMG should not be expected to show identical post-fatigue patterns. In the present study, lower post-fatigue IEMG indicates a smaller amplitude–time integral of the unnormalized EMG envelope during the upward lifting phase, but does not necessarily indicate lower muscle force or fewer recruited motor units. Several non-mutually exclusive explanations are possible. Fatigue may have reduced the level of muscle excitation that participants could sustain during the post-fatigue lifts; excitation may have been redistributed across muscles or among different regions of the same muscle; or changes in posture, movement coordination, muscle length and velocity, and passive tissue contributions may have altered the excitation required from the recorded superficial muscles. Because lifting time was similar or slightly longer after fatigue, a shorter integration period is unlikely to be the primary explanation, suggesting that a lower mean EMG-envelope amplitude contributed to the lower IEMG. The concurrent decrease in MF supports the presence of a fatigue-related electrophysiological change but does not identify the specific mechanism responsible for the lower IEMG. Future studies should analyze phase duration together with mean and peak EMG amplitude, record a broader set of superficial and deep muscles, and combine EMG with muscle-force estimation to distinguish among these explanations.

### 4.3 Practical implications for athletic training and lifting performance

HS and DS showed relatively smaller lumbar excursions than FB, but HS did not consistently reduce net generalized lumbar joint moments across all segments. Thus, neither technique should be regarded as universally protective, and the present data do not establish differences in internal spinal loading or lower-extremity load redistribution. Fatigue-related changes in peak-moment timing and IEMG may be relevant to movement consistency and fatigue management during lifting practice, but they do not support specific recommendations regarding spinal-load reduction or injury prevention.

### 4.4 Methodological considerations and future directions

Despite the integrated use of kinematic, kinetic, and electromyographic analyses together with OpenSim-based inverse kinematics and inverse dynamics, several limitations should be acknowledged. The OpenSim analysis used a scaled generic FBLS model, experimental marker trajectories, ground reaction forces, and estimated external box forces to calculate lumbar kinematics and net generalized segmental joint moments. These outputs may be influenced by scaling assumptions, marker-placement and kinematic-tracking errors, and uncertainty in the applied external forces. No Static Optimization, Computed Muscle Control, muscle-force estimation, joint-reaction analysis, or other muscle-actuator-based simulation was performed. Therefore, the analysis did not estimate muscle mechanical properties, individual muscle forces, spinal compression or shear forces, or tissue-specific spinal loads. In addition, surface electromyography primarily reflects the activity of superficial muscles and is susceptible to factors such as electrode placement, subcutaneous tissue thickness, and signal crosstalk. As a result, the observed reductions in muscle activation are more likely to reflect adjustments in superficial muscle activity and may not fully represent changes in the overall neuromuscular control of the trunk. Because no separate maximal-strength or 30-kg lifting-capacity test was administered, the findings should be interpreted within the selected study sample and should not be generalized to untrained workers or used to define a safe occupational lifting limit.

The fatigue protocol adopted in this study was based on a short-duration, controlled laboratory task, which differs from the prolonged and multifactorial fatigue typically experienced in real-world manual handling scenarios. Although self-selection avoided imposing one of the three experimental techniques during fatigue induction, unclassified individual variation in lifting strategy may have influenced which muscle groups were most affected and reduced the sensitivity to detect technique-specific Pre–Post effects. In practice, fatigue often coexists with cognitive demands, task variability, and environmental perturbations, potentially leading to more complex adaptations in movement strategy. Therefore, the present findings regarding fatigue-related changes in peak-moment timing and IEMG of the recorded muscles should be interpreted with caution when applied to occupational settings. Future research may benefit from incorporating high-density electromyography or imaging techniques to better capture deep muscle activity, as well as from applying subject-specific modeling or finite element approaches to improve the physiological accuracy of spinal load estimation. Extending investigations to longer-duration tasks and more complex environments may also provide further insight into the cumulative effects of fatigue on movement strategies. Integrating biomechanical analyses with injury surveillance or intervention studies could further strengthen the translation of these findings into practical applications.

## 5 Conclusion

This study examined the effects of different lifting techniques and fatigue on lumbar biomechanics and neuromuscular responses using OpenSim-based inverse kinematics and inverse dynamics analyses together with independent surface-EMG analysis. The results showed that lifting technique influenced the measured lumbar kinematics, net generalized segmental joint moments, and IEMG of the recorded muscles. FB was associated with greater lumbar flexion and higher semitendinosus IEMG, whereas HS and DS showed relatively smaller lumbar excursions. However, HS did not consistently result in lower net generalized lumbar joint moments across all segments and therefore showed an intermediate net-moment pattern rather than a uniformly lower profile.

Fatigue did not produce a clear increase in the measured net generalized lumbar joint moments. Instead, it was accompanied by delayed peak-moment timing and lower IEMG in several recorded muscles. These observations describe fatigue-related differences in the measured temporal and EMG-derived variables, rather than directly demonstrating altered spinal stability, internal spinal loading, or a specific neuromuscular coordination mechanism. From an applied perspective, lifting performance may depend not only on technique selection, but also on the ability to maintain consistent movement execution under fatigue.

Overall, lifting technique was associated with distinct lumbar kinematic and net generalized segmental joint-moment patterns, whereas fatigue was primarily reflected in peak-moment timing and the IEMG of the recorded muscles.

## Data Availability

The raw data supporting the conclusions of this article will be made available by the authors, without undue reservation.
