# Supplementary material for: Effects of fatigue on lumbar biomechanics and muscle activation across different lifting techniques: an OpenSim-based analysis
Source: Front Bioeng Biotechnol. 2026 Sep 29;14:1898319. doi: 10.3389/fbioe.2026.1898319 (PMC13658470; doi:10.3389/fbioe.2026.1898319)
Supplement: Supplementary file 1 [file Table1.docx]

**Supplementary Table S1**

Detailed description of the customized 39-marker whole-body configuration and four box markers.

| **Marker label** | **Segment** | **Anatomical location** | **Purpose** |
| --- | --- | --- | --- |
| LFHD | Head | Left temple region | Head tracking |
| RFHD | Head | Right temple region | Head tracking |
| LBHD | Head | Left posterior head region | Head tracking |
| RBHD | Head | Right posterior head region | Head tracking |
| LSHO | Shoulder | Left acromion process | Shoulder position tracking |
| RSHO | Shoulder | Right acromion process | Shoulder position tracking |
| C7 | Trunk | Spinous process of the seventh cervical vertebra | Upper-trunk tracking |
| T10 | Trunk | Spinous process of the tenth thoracic vertebra | Thoracic-segment tracking |
| CLAV | Trunk | Clavicle-sternum junction | Anterior upper-trunk tracking |
| STRN | Trunk | Lower sternum/xiphoid region | Anterior trunk tracking |
| RBAK | Trunk | Mid-region of the right scapula | Thoracic-segment tracking |
| LASI | Pelvis | Left anterior superior iliac spine | Pelvic position and orientation |
| RASI | Pelvis | Right anterior superior iliac spine | Pelvic position and orientation |
| LPSI | Pelvis | Left posterior superior iliac spine | Pelvic position and orientation |
| RPSI | Pelvis | Right posterior superior iliac spine | Pelvic position and orientation |
| LKNE | Thigh | Left lateral femoral epicondyle | Knee-joint localization |
| RKNE | Thigh | Right lateral femoral epicondyle | Knee-joint localization |
| LFP3 | Thigh | Distal third of the left thigh | Technical tracking of the left thigh |
| RFP2 | Thigh | Mid-region of the right thigh | Technical tracking of the right thigh |
| LANK | Shank | Left lateral malleolus | Ankle-joint localization |
| RANK | Shank | Right lateral malleolus | Ankle-joint localization |
| LTP2 | Shank | Mid-region of the left shank | Technical tracking of the left shank |
| RTP3 | Shank | Distal third of the right shank | Technical tracking of the right shank |
| LHEE | Foot | Posterior midpoint of the left heel | Rearfoot tracking |
| RHEE | Foot | Posterior midpoint of the right heel | Rearfoot tracking |
| LTOE | Foot | Distal region of the left first toe | Forefoot tracking |
| RTOE | Foot | Distal region of the right first toe | Forefoot tracking |
| LUPA | Upper arm | Mid-region of the left upper arm | Technical tracking of the left upper arm |
| RUPA | Upper arm | Mid-region of the right upper arm | Technical tracking of the right upper arm |
| LELB | Elbow | Left lateral humeral epicondyle | Elbow-joint localization |
| RELB | Elbow | Right lateral humeral epicondyle | Elbow-joint localization |
| LFRA | Forearm | Mid-region of the left forearm | Technical tracking of the left forearm |
| RFRA | Forearm | Mid-region of the right forearm | Technical tracking of the right forearm |
| LWRA | Wrist | Radial side of the left wrist | Wrist-joint localization |
| RWRA | Wrist | Radial side of the right wrist | Wrist-joint localization |
| LWRB | Wrist | Ulnar side of the left wrist | Wrist-joint localization |
| RWRB | Wrist | Ulnar side of the right wrist | Wrist-joint localization |
| LFIN | Hand | Base of the left index finger | Hand tracking |
| RFIN | Hand | Base of the right index finger | Hand tracking |
| B1 | Box | First non-coplanar corner of the test box | Box position and orientation tracking |
| B2 | Box | Second non-coplanar corner of the test box | Box position and orientation tracking |
| B3 | Box | Third non-coplanar corner of the test box | Box position and orientation tracking |
| B4 | Box | Fourth non-coplanar corner of the test box | Box position and orientation tracking |

**Note.** The first 39 markers were attached to the participant; B1–B4 were attached to four non-coplanar corners of the test box.
